# Reply to Dr. Hopkins

**Published:** 1900-12

**Authors:** W. D. Miller

**Affiliations:** Victoriastr. 30, Berlin


### Foreign Correspondence.

### REPLY TO DR. HOPKINS.

TO THE EDITOR:

SIR,—In the October number of your esteemed journal, page 708, will be found a statement by Dr. Hopkins to the effect that he has succeeded in producing artificial caries by a pure culture of a mouth-bacterium, and believes it to be the first authentic instance in which this has been accomplished. I have already repeatedly called attention to the fact that artificial caries was produced, by means of a pure culture of a bacterium isolated from carious dentine, as early as 1884, and that the experiments were described in the *Independent Practitioner* for 1884, page 113 *et seq.*

Yours very sincerely,

W. D. MILLER.

VICTORIASTR. 30, BERLIN, October 19, 1900.

